# Detection of arousal and valence from facial expressions and physiological responses evoked by different types of stressors

**DOI:** 10.3389/fnrgo.2024.1338243

**Published:** 2024-03-15

**Authors:** Juliette Bruin, Ivo V. Stuldreher, Paola Perone, Koen Hogenelst, Marnix Naber, Wim Kamphuis, Anne-Marie Brouwer

**Affiliations:** ^1^TNO Human Factors, Netherlands Organization for Applied Scientific Research, Soesterberg, Netherlands; ^2^Experimental Psychology, Helmholtz Institute, Faculty of Social and Behavioral Sciences, Utrecht University, Utrecht, Netherlands; ^3^Artificial Intelligence, Donders Centre, Faculty of Social Sciences, Radboud University, Nijmegen, Netherlands

**Keywords:** facial expression, skin conductance, heart rate, heart rate variability, arousal, valence, stress, machine learning

## Abstract

Automatically detecting mental state such as stress from video images of the face could support evaluating stress responses in applicants for high risk jobs or contribute to timely stress detection in challenging operational settings (e.g., aircrew, command center operators). Challenges in automatically estimating mental state include the generalization of models across contexts and across participants. We here aim to create robust models by training them using data from different contexts and including physiological features. Fifty-one participants were exposed to different types of stressors (cognitive, social evaluative and startle) and baseline variants of the stressors. Video, electrocardiogram (ECG), electrodermal activity (EDA) and self-reports (arousal and valence) were recorded. Logistic regression models aimed to classify between high and low arousal and valence across participants, where “high” and “low” were defined relative to the center of the rating scale. Accuracy scores of different models were evaluated: models trained and tested within a specific context (either a baseline or stressor variant of a task), intermediate context (baseline and stressor variant of a task), or general context (all conditions together). Furthermore, for these different model variants, only the video data was included, only the physiological data, or both video and physiological data. We found that all (video, physiological and video-physio) models could successfully distinguish between high- and low-rated arousal and valence, though performance tended to be better for (1) arousal than valence, (2) specific context than intermediate and general contexts, (3) video-physio data than video or physiological data alone. Automatic feature selection resulted in inclusion of 3–20 features, where the models based on video-physio data usually included features from video, ECG and EDA. Still, performance of video-only models approached the performance of video-physio models. Arousal and valence ratings by three experienced human observers scores based on part of the video data did not match with self-reports. In sum, we showed that it is possible to automatically monitor arousal and valence even in relatively general contexts and better than humans can (in the given circumstances), and that non-contact video images of faces capture an important part of the information, which has practical advantages.

## Introduction

Screenings and assessments for high-risk professions (e.g., police, military) often include questionnaires and interviews. However, research has shown that self-assessment questionnaires and assessments of recruiters or employers are often biased, unreliable or incorrect (Donaldson and Grant-Vallone, 2002; Luxen and Van De Vijver, 2006; Huffcutt et al., 2013; Kappen and Naber, 2021). When filling in self-assessment questionnaires, candidates tend to bias their responses based on their perception of the expectations of the assessor, pretending to be better or different than they really are (Donaldson and Grant-Vallone, 2002). Similarly, despite training and good intentions, also the evaluations from Human Resources experts are not immune to biases. In fact, it has been shown that interviewers can be implicitly biased by the gender, the ethnicity and the physical appearance of the candidate (Luxen and Van De Vijver, 2006; Riach and Rich, 2010; Zschirnt and Ruedin, 2016). Hence, objective measurements are necessary to remove potential implicit biases from the interview process when evaluating whether there is a good fit between an individual's characteristics and the demand of the offered job. This may be particularly true when assessing stress resilience in candidates for high-risk professions. They are frequently exposed to stressful and challenging circumstances and need to be stress resilient to recover rapidly from these situations to maintain health (Thayer et al., 2012).

One development in Human Resources entails a shift toward the use of game-based performance assessments by recruiters (Bina et al., 2021). Furthermore, candidates' behavior (notably, facial expression) and physiological activity during job interviews may be used as an objective, reliable measure for candidate selection (Kappen and Naber, 2021; Kuipers et al., 2023). The two tools could be combined in that assessment games can offer a variety of stressful settings during which physiological and behavioral activity can be recorded. Given that stress is characterized by high arousal and negative valence (Kuppens et al., 2013), the measurement of arousal and valence would be useful to investigate individuals' responses to potentially stressful situations.

Various physiological systems, including the autonomic nervous system (ANS), respond to stressors (Kemeny, 2003) in order to help organisms to maintain homeostasis (i.e., the effort of an organism to keep its physiological parameters within an acceptable range despite environmental changes; von Holst, 1998). Given that the ANS is operating outside individuals' awareness and its activation is influenced by individuals' affective states, measuring ANS activity provides an unbiased tool to investigate reaction to and resilience in stressful situations. The ANS has two major branches: the sympathetic nervous system (SNS), mainly associated with the “fight-or-flight” response, and the parasympathetic nervous system (PNS), mainly associated with the “rest and digest” response. Increased activation of the SNS indicates higher arousal states. Measures derived from electrocardiogram (ECG—heart rate and heart rate variability, i.e., variation in the time interval between heartbeats and the extent to which heart rate is tuned to respiration), reflect the balance between the regulation of the SNS and PNS branches of the autonomic nervous system (McCorry, 2007; Jongyoon et al., 2012). Electrodermal activity (EDA, also referred to as skin conductance) reflects the activity of the sweat glands and is solely regulated by the SNS (Posada-Quintero and Chon, 2020). It is particularly sensitive to high arousal states (e.g. fear, anger, stress; Lazarus et al., 1963; Dawson et al., 2007). ECG and EDA measures can provide important insight into how individuals perceive and respond to their environment and to different stressors. For example, Toet et al. (2017) found that military personnel showed weaker responses in EDA and heart rate to a controlled stressor than civilians, suggesting that such responses are associated to stress resilience in general.

While ECG and EDA measures reflect individuals' arousal level in response to contextual and environmental changes, the link between these measures and individuals' level of valence is less clear. Facial expression may be a more reliable measure to assess valence. Various studies showed that facial features, expressions and motions as recorded via cameras and analyzed using AI are informative of mental state (Hoque et al., 2012; Sharma and Gedeon, 2012; Pedrotti et al., 2014; Hoegen et al., 2019; Kappen and Naber, 2021; Kuipers et al., 2023). Particularly, contrary to the long-lasting view that facial expressions convey fixed, generalizable emotion states (Ekman, 1984), it has been proposed that they serve as indices of individuals' affective state, indicating points on the dimensions of valence and arousal as a consequence of internally or externally changing events (Barrett, 2006). However, it is still unclear whether video measures are informative on stress (valence and arousal) in spontaneous behavior displayed in several contexts of interest. In fact, it seems reasonable that facial expressions are strongly influenced by the context in which they are displayed. For example, one would expect quite different facial expressions during social tasks, where facial expression serves a communication purpose, and cognitive tasks, where this is not the case. Additionally, it has been shown that individuals vary considerably with respect to facial expressiveness. Moreover, only few studies exploit the dynamic nature of facial expressions, while it has been demonstrated that motion is an important feature to identify subtle facial expressions (Ambadar et al., 2005). We think it is important to use dynamic facial expressions to evaluate arousal and valence responses that are spontaneously evoked in different contexts and different individuals.

Given the range of possible measures, it is important to evaluate to what extent we can use them to detect stress-related mental state. ECG and EDA measures are associated with stress in a more straightforward, biologically explainable way than facial expression. Therefore, physiological measures of arousal can be expected to be more consistent across participants and contexts than recorded facial expression (Barrett et al., 2019). However, the need of using sensors to record such physiological data prohibit or complicate setups, certainly when usage is envisioned at home. On the other hand, video measures, which would not only include facial expression, but also head pose and gaze, can be a better measure of valence, would be convenient, cheap and could be employed also during online interviews and online game-based assessment.

In the current study, we aim to build models that can detect stress (operationalized by arousal and valence) across individuals and across contexts on the basis of video data (facial expression, head pose and gaze), physiological data (ECG and EDA) and a combination of the two. Specifically, we induce stress in different ways (varying context) and add baseline conditions that were especially designed to differ only from the stressor conditions in terms of elicited arousal and valence, in order to obtain a varying range of experienced mental states. The ground truth that we use is self-report (high-low arousal, and high-low valence according to a chosen absolute cut-off, where we choose the middle of the scale), rather than judgements of experts (as often done before, e.g., in Dinges et al., 2005; Sutherland et al., 2015). We think that in the experimental situation that we test, i.e., a situation in which it is clear that individuals' answers are confidential and will not have consequences in their life, introspective judgements are more reliable than observers' judgement. Models are trained and tested within context (i.e., one type of stressor, with or without accompanying baseline), as well as across contexts (i.e., across multiple types of stressors and baselines). Model performance and feature importance are examined. We expect that estimating mental state within a certain narrow context may work relatively well for video measures, but that when it comes to generalization across contexts, physiological features are especially important. We expect that physiological features match better with arousal than valence, whereas video measures may be relatively suitable to estimate valence. To get an impression of the conspicuity of arousal and valence information in the face to humans, we roughly compare results of models to human expert judgements.

Our dataset (available at https://osf.io/ztbek/) adds to previous datasets that contain affective responses recorded through self-report, video and physiological sensors (Koelstra et al., 2011; Soleymani et al., 2011; Miranda-Correa et al., 2018) in that we did not only use movies to affect valence and arousal in our participants, but also other tasks. This enables testing for generalizability of models across contexts.

## Materials and methods

### Participants

Fifty-one participants (25 male, mean age = 38, SD = 13.49) took part in the experiment. They were recruited from the participant pool of the research institute where the study took place (TNO). People with visual and auditory impairments or psychological problems (for which in treatment within the previous year) could not participate. All participants signed a form of informed consent before the start of the experiment and received monetary compensation after completing the experiment. The study was approved by the Internal Review Board at TNO (reference number 2022-093). Two participants were excluded from the analysis: for one participant the time log data was not saved correctly, the other participant consistently provided the same valence and arousal scores for all tasks.

### Materials

Video recordings of the participant's face were captured with a webcam (Microsoft LifeCam HD-3000) and recorded through OBS Studio. Electrocardiogram (ECG) and electrodermal activity (EDA) were recorded using the BioSemi ActiveTwo AD-box. EDA electrodes were placed on the fingertips of the index and middle finger of the non-dominant hand. ECG electrodes were placed on the lowest floating left rib and the right clavicle. In addition, heart rate and EDA were simultaneously recorded using a Polar H10 chest strap and an EdaMove4 with self-adhesive electrodes on the palm of the non-dominant hand. Data from these recordings are not discussed in the present manuscript.

Participants filled out the Perceived stress scale (Cohen et al., 1994), the 10-question Connor-Davidson Resilience Scale (Connor and Davidson, 2003) and the short Big Five Inventory 2 (Soto and John, 2017). Two visual analog scales (VAS) running from 0 to 10 were used to capture the experienced valence (marked “unpleasant” at the left and “pleasant” at the right) and arousal (marked “calm/relaxed/bored” at the left and “stressed/energetic/excited” at the right) after each task. All questionnaires were filled out through Survalyzer, an online survey application.

The experimental tasks were embedded in a browser application developed by Neurolytics (Utrecht, The Netherlands). During the experimental tasks, the application collected task-related data in addition to webcam recordings. The experiment was run on a 14-inch laptop (Dell Latitude E7250). The laptop was additionally used to send out markers for aligning all different data sources. The laptop was connected to a 27 inch monitor (Samsung Syncmaster) and participants controlled the laptop with the use of a separate keyboard and a mouse (Dell). A different laptop was used to record the signals from the BioSemi.

### Stimuli and design

**Figure 1:**
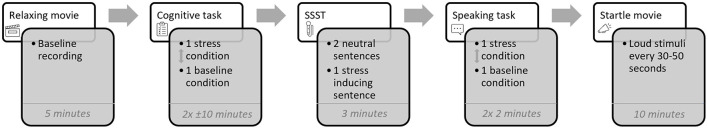
Schematic overview of the sequence and duration of experimental conditions conducted by each participant. The order of the different task blocks was fixed, while the arrangement of baseline and stressor conditions varied for the cognitive and speaking tasks (as indicated by the up and down pointing arrows). The orderings of conditions were counterbalanced across participants.

**Figure 2:**
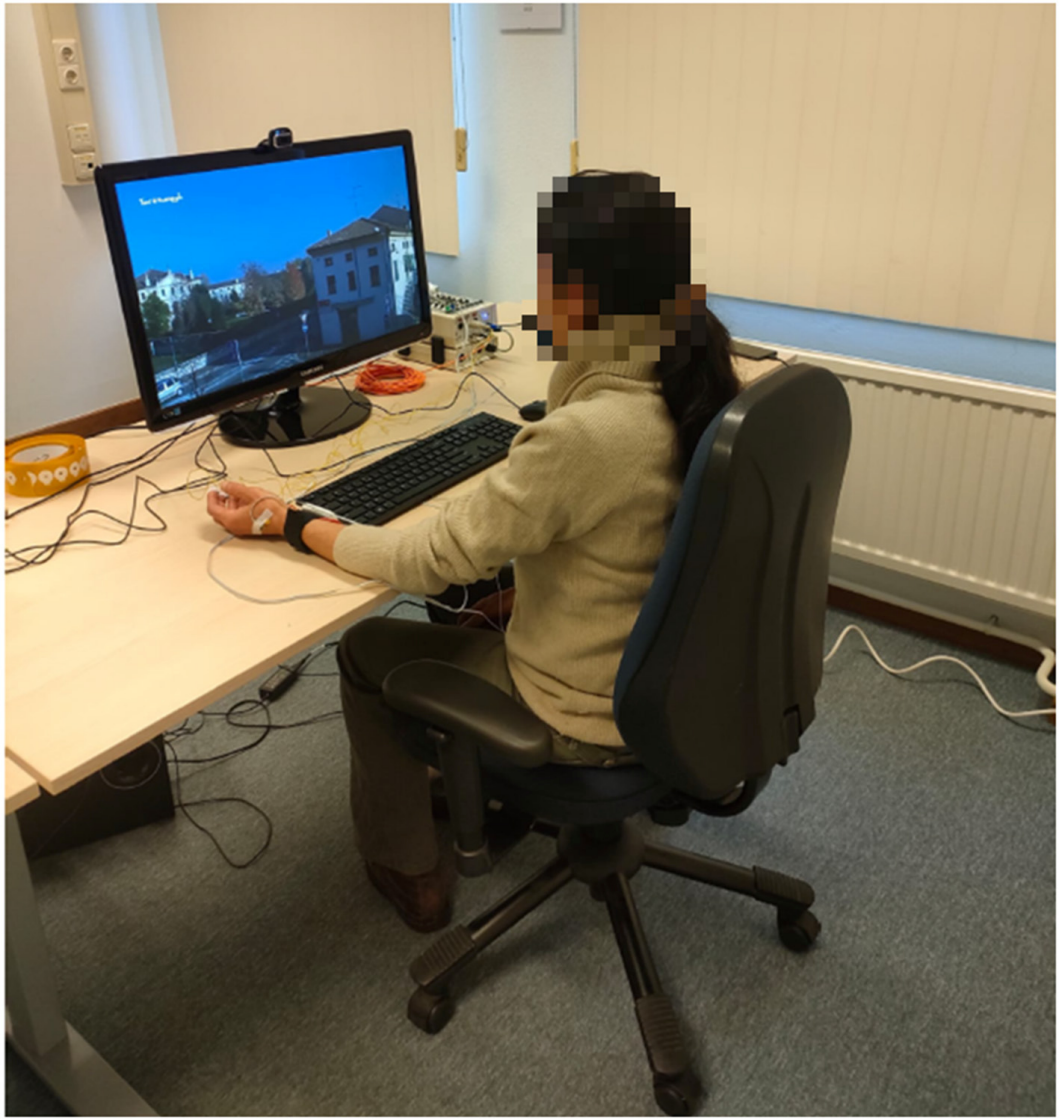
Participant in the setup, watching the relaxing movie.

Figure 1 shows the schematic overview of the experiment. Each participant performed successively a relaxing movie condition (baseline recording), a cognitive task (baseline and stressor), the sing-a-song stress test (involving a baseline and stress interval), a speaking task (baseline and stressor), and a startle movie condition. The order of these five conditions was fixed; the order of baseline and stressor parts were varied for the cognitive task and the speaking task. The resulting four different condition orders were counterbalanced across participants. Figure 2 shows a participant in the setup.

#### Relaxing movie—Baseline recording

To assure all participants were at ease and familiar with the setup, a 5-min neutral movie consisting of scenes of an Italian village was watched (“Pietraperzia” validated as neutral in Maffei and Angrilli, 2019; the spoken Italian text was replaced by calm music).

#### Cognitive task

The cognitive *stressor* task consisted of a mental capacity test with 40 multiple choice questions, each accompanied by four answer options. Questions were presented in four blocks of 10 questions, with each block assessing a different cognitive skill: general knowledge, logical reasoning, numerical reasoning and verbal reasoning. Participants were instructed to answer all questions as good and quickly as possible using the mouse within 2.5 min per block. Questions were designed such that most participants would not be able to answer all questions within 2.5 min. A conspicuous count-down timer was visible on the screen and the timer font changed from black to red during the last 10 s to increase the time pressure. After 2.5 min a block was terminated and a new block started after a 10-s break. The *baseline* version consisted of a questionnaire that was matched for the number of questions, the length of the questions and the number of answering options. However, the questions were rewritten such that the cognitive demand was extremely low and such that answering took about the same time (e.g., “Wait approximately 15 seconds and select the second answer” or “Hover over the questions and select answer C”). The count-down timer was also visible during the baseline measurement but participants were explained that it was not the aim to answer as quickly as possible.

#### Sing-a-song stress test

In this implementation of the Sing-a-song stress test (SSST; Brouwer and Hogervorst, 2014), participants were presented with two neutral sentences followed by a 60-s countdown. The third sentence that appeared instructed participants to sing a song after the countdown had reached zero. This last 60-s countdown interval is the *stressor* which is compared to the preceding 60-s *baseline* countdown interval.

#### Speaking task

During the *stressor* version of this task, participants were instructed to verbally reply over the course of 1 min with a thoughtful moral judgement on a moral dilemma that would be presented to them, and that their video-recorded response would be reviewed by an expert. They subsequently viewed a 1-min movie of a sergeant asking the participant how they would deal with noticed, strictly forbidden drug usage of a colleague in the military. Participants then responded for about a minute. In the *baseline* version, participants were instructed to verbally respond to a movie, and that their response would serve as a baseline which would not be reviewed. The 1-min movie for this baseline recording showed a man telling about him just having bought a wheelbarrow at a supply store. At the end of the movie, the man asked the participant to tell about their day, which participants did during the minute thereafter.

#### Startle movie

Participant watched a suspenseful 10-min clip cut from the TV Mini Series “The Haunting of Hill House” (Episode 9 “Screaming meemies”, 38:10-48:34) in which sounds were normalized to 60 dB. Loud (95 dB) 50 ms white-noise stimuli were superimposed with 30–50 s intervals between them. All participants viewed and heard the exact same edited movie clip.

### Procedure

The participants were informed about the experiment without going into detail of the conditions—they were told they would be asked to watch short movies, to answer questionnaires and to perform other small tasks. After the sensors were attached they filled out the Short Big Five Inventory, Connor-Davidson Resilience Scale and the Perceived Stress Scale. Then they went through the conditions as described under stimuli and design. After each condition (baseline recording movie, cognitive task–stressor, cognitive task–baseline, SSST, speaking–stressor, speaking-baseline and startle movie), they filled out the VAS arousal and the VAS valence. For the SSST they filled these out twice; once for the baseline- and once for the stressor-interval. Thus, for each participant, we obtained eight arousal and eight valence scores.

### Analysis

#### Feature extraction

In total, 65 features were extracted from the video data and 44 from the physiological data. A full overview of these features can be found in Tables A1, A2.

##### Camera

The Webcam recordings were cut and saved per task in.avi format without sound. Features for all videos were extracted with the FeatureExtraction function from OpenFace 2.2.0. (Baltrusaitis et al., 2018). This toolbox calculates a range of features related to gaze, head pose, and 17 facial action units for each frame. These action units are based on the Facial Action Coding System (FACS), which was developed to encode different movements and behaviors of the face and eyes (Ekman and Friesen, 1978; examples can be viewed at https://imotions.com/blog/learning/research-fundamentals/facial-action-coding-system/). Based on recommendations by the developer of OpenFace (Baltrusaitis, 2017), for all facial features, intervals with a confidence score (as calculated by OpenFace) below 70% were not included in further analysis. Single feature values per participant and per interval of interest (relaxing movie, startle movie, and the baseline and stress intervals of each of the three tasks) were obtained using means, variance over time, and degree of feature presence. All features were scaled with use of the Python StandardScaler function from scikit-learn.

##### ECG

R-peaks were detected in ECG with the use of a detection algorithm based on Pan and Tompkins (1985). Inter-beat intervals were determined and converted to heart rate. Heart rates below 30 bpm or above 200 bpm were discarded; deviations from the mean, greater than three standard deviations were discarded in an iterative process with three iterations. Mean, minimum, maximum, standard deviation, area under the curve, kurtosis and skewness of the IBI values were determined as single values per participant and per interval of interest (relaxing movie, startle movie, and the baseline and stress intervals of each of the three tasks). In addition, we determined different measures of heart rate variability [power in the very low frequency (VLF, 0–0.04 Hz), low frequency (LF, 0.04–0.15 Hz), high frequency (HF, 0.15–0.40 Hz) domains; the ratio between the power in the LF and HF bands; root mean square of successive differences (RMSSD)]. For spectrum computation inter-beat intervals were interpolated onto a regularly sampled time series at a frequency of 5 Hz prior to calculating HRV frequency parameters. The length of the signal used to derive statistical features matched the duration of the task, as indicated in Figure 1. All features were scaled with use of the Python StandardScaler function from scikit-learn.

##### EDA

EDA signals were decomposed into tonic and phasic components using the Ledalab toolbox (Benedek and Kaernbach, 2010). Mean, standard deviation, minimal and maximum amplitude, the area under the curve (AUC), kurtosis and skewness were determined for the full signal and the phasic component. Again, the signal length of the intervals of interest, from which the statistical features were calculated, matched the duration of the task, as depicted in Figure 1. All features were scaled with use of the Python StandardScaler function from scikit-learn.

#### Data descriptives

Before modeling, we explored the overall effects of the different conditions on the variables. Wilcoxon signed-rank tests were performed on the self-reported valence and arousal scores as well as on all features to determine whether differences between corresponding baseline and stress tasks were significant. The number of “high” (>5) and “low” ( ≤ 5) arousal and valence responses was counted for each of the conditions. Modeling is only possible with a sufficient number of both “high” and “low” responses to prevent bias to predict a specific task (and thus its predominant label) rather than the experienced mental state. In addition, Kruskal-Wallis H tests were used to determine which features showed significant differences between “low” and “high” self-reported arousal and valence.

#### Modeling

In order to explore whether self-reported valence and arousal could be predicted from video and/or physiological data, logistic regression models were trained on video features, or physiological features (EDA and ECG), or both. To examine generalization of features across contexts, three subsets of conditions were used as input of different models. In the specific context models, data from only one condition was included (baseline recording movie, startle movie, either the baseline or the stressor condition of the cognitive task, public speaking task or SSST). In the intermediate context models, both the baseline and stressor condition of one task type were included. In the general context models, data from multiple task conditions, were used as input for the models.

For each individual model, 90% of the dataset was used as training and validation data (44 participants) while the remaining 10% was kept apart completely for testing (five participants), following recommendations on proportions of training and validation, and test data (Panicker and Gayathri, 2019; Sharma et al., 2021). Participants were randomly distributed over the train and test set. Each instance represented one task for a specific participant. The model did not have access to the task and participant identifiers to make the model task and subject independent. For validation and optimization of the models, the full training set was used because of the limited amount of data—the remainder of this section description below applies to the data without the five participants that were kept apart.

**Figure 3:**
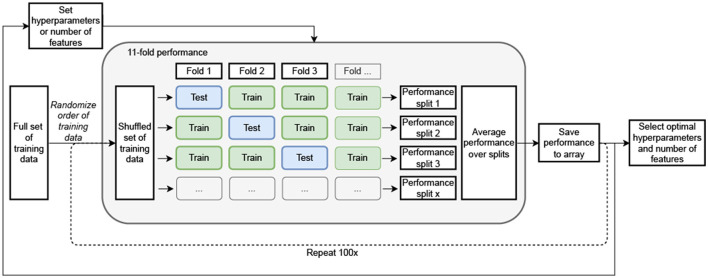
Schematic overview of optimal hyperparameter, feature amount and model selection. The optimal settings were identified by averaging performance across multiple iterations of randomized 11-fold cross-validation on the training data. In each validation iteration, a unique fold was chosen as the test set, while the models were trained on the remaining 10-folds. This process was repeated 100 times to cover all possible combinations of training and test data splits from the available dataset.

To reduce the chance of overfitting, minimum redundancy—maximum relevance (MRMR) (Ding and Peng, 2005) was applied to decrease the number of features from 109 to 40. Next, the optimal number of features was determined by the average performance on iterations of randomized 11-folds of the training data (Figure 3). For each validation iteration, a different fold was selected as the test set and the algorithms were trained on 10 of these folds, followed by a test on the remaining fold. All folds were selected randomly based on participants. This process was repeated 100 times to include all possible combinations of training and test data from the available training data. The performance of the models utilizing 1, 3, 5, 10, 20, 30, 40, and all (109) features were compared and the best option was selected. The corresponding precision, recall and F1-scores were checked to make sure the model did not highly favor one specific category. Based on these outcomes, the optimal number of features was selected for the final models.

To optimize the models and reduce the chance of overfitting, hyperparameter tuning was performed on the training set. Here, the training set was again split into 11-folds (Figure 3) but for this step, random instances were selected instead of participants to further reduce the chance of overfitting. Again 100 repetitions of random fold selection were performed. Grid search was used to find the optimal hyperparameters for all individual models. Moreover, before training, the classes were balanced with use of the SMOTE (synthetic minority oversampling technique) function from scikit-learn.

Finally, to determine the final model performance, the optimized number of features and hyperparameters were used.

#### Analysis of modeling results

For all classification models, performance was assessed by examining accuracy, precision, recall and F1-scores. Accuracy, precision, recall and F1-scores were determined per class and over classes, with the use of macro averaged scores. Both the performance on the test set and the training set (by averaging the performance on the training set with the use of the same cross-validation and averaging approach as described for algorithm selection and feature selection) were assessed. Because precision, recall and F1 showed similar results as accuracy scores, and did not suggest that accuracy scores were strongly affected by the majority class, we report the more intuitive measure of accuracy. In traditional machine learning, typically only the performance on the test set would be considered. However, because of the limited test set (five individuals), we focus on the cross-validation results from the training set. Earlier, similar papers did not include a test set at all, and only evaluated results through cross-validation in the training data.

#### Chance performance

To facilitate interpretation of the models' performance, we assessed whether the models performed better than would be expected by chance with the use of permutation testing using the same settings of the models, the same data and the same class imbalance, only, the “high” and “low” valence and arousal labels were shuffled. All models were trained and tested on randomized labels. This process was repeated 10,000 times to create a chance distribution. Separate processes were created for the training and test settings, keeping the basic settings like iterations, splits and oversampling consistent with the normal modeling approach. For each model, the model performance was then compared to the created distribution. A *p*-value was established by dividing the number of occurrences in the distribution that outperformed the true model and dividing this count by the total number of repetitions (10,000). Note that similar accuracies for different models can render different (significant and non-significant) *p*-values because of the difference in data distribution and the variance in random patterns between sets.

#### Feature contribution

As described above, and as formulated as one of our main questions, we examined performance of models as a function of the type of features used (video, physio, or video-physio). In addition, we examined more closely which features were selected in the various models based on video-physio features, and the extent to which the same features were used in different models.

### Expert assessments

To get an impression of the performance of the model in relation to human observation, performance of expert observers was determined. Three selection psychologists came to the lab to rank different webcam recordings from the experiment. For each recording, the experts were asked to rate the participant's experienced valence and arousal on a VAS scale for valence and arousal. The scales were identical to the scales that were filled out by participants during the experiment.

The experts watched and rated the videos through PsychoPy^®^. Videos were retrieved for the cognitive, public-speaking and SSST tasks. Because of time limitations, each video was shortened to 10 s, starting 2 s after the start of each of the conditions, therewith capturing the first reaction to each stressor task, and the equivalent in the baseline. All videos were muted. Each expert rated videos from all three tasks and for all participants, such that every rating of every participant in the cognitive, public-speaking and SSST baseline and stress tasks was matched with ratings of three observers. The videos were clustered per participant and per task (cognitive/public speaking/SSST). The presentation order of participants and the stressor/baseline variants were randomized. Since observers could (learn to) guess the task that the participant was exposed to from watching the video, the name of the task (cognitive/public speaking/SSST) was displayed to reduce variation in available information across time and across observers.

Fleiss Kappa scores were calculated to capture the inter-rater agreement. Furthermore, it was assessed whether the expert observers performed better rating arousal and valence “high” or “low” than would be expected by chance. The same analysis was done as described above for the participants' ratings: all labels were shuffled and compared to the true labels. Again, this process was repeated 10,000 times to establish a chance distribution. *P*-values were calculated by summing the number of instances above expert performance and dividing it by 10,000.

## Results

### Descriptives

**Figure 4:**
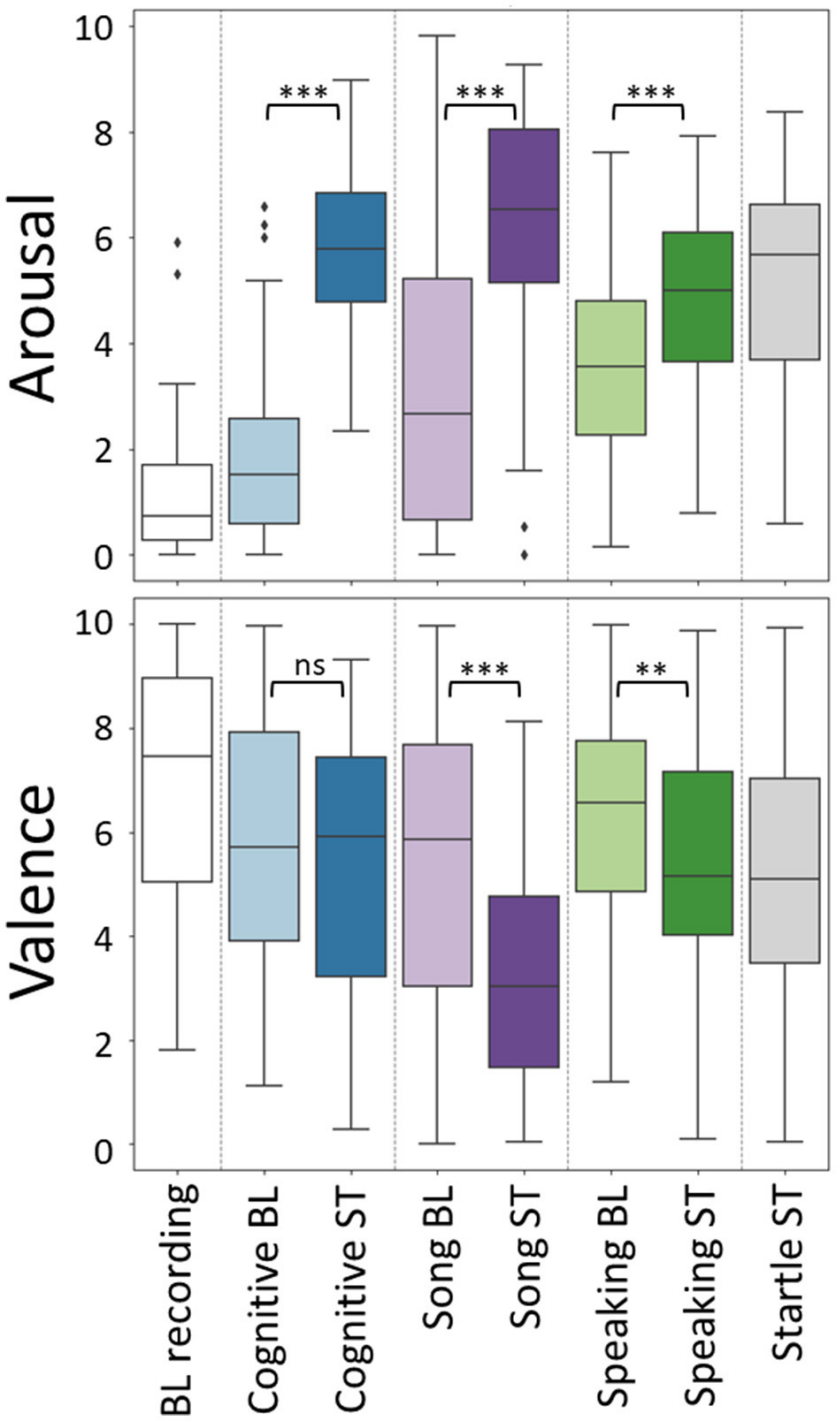
Self-reported arousal **(top)** and valence **(bottom)** per condition, as self-reported on a VAS scale running from 0 (low) to 10 (high). Results of Wilcoxon signed ranks tests, comparing between the baseline (BL) and stress (ST) conditions for each of the three tasks are indicated; ** and *** indicate significance levels of 0.01 and 0.001, respectively, ns refers to not significant.

Figure 4 shows self-reported arousal and valence for each of the eight conditions. Wilcoxon signed ranks tests were performed to compare the self-reported arousal and valence between the baseline and stress conditions for each of the three tasks. For arousal, all of these tests were significant, indicating higher arousal in the stressor compared to the baseline task (cognitive task: W = 4.0, *p* < 0.001; SSST: W = 101.5, *p* < 0.001; public speaking: W = 174.0, *p* < 0.001). For valence, baselines were judged to be more pleasant than stressors for SSST (W = 200.0, *p* < 0.001) and public speaking: W = 311.5, *p* = 0.004) but there was no effect for the cognitive task (*W* = 492.5, *p* = 0.449). The box plots show a large variation between participants, spanning almost the entire scale for many of the conditions, in particular for valence.

We examined the number of “low” and “high” arousal and valence values (i.e., lower or higher than 5, representing the center of the VAS scale) in the different conditions. Except for the arousal labels of the baseline recording and the baseline of the cognitive task, all classes contain at least 10 instances of each “low” and “high” label, enabling modeling. Table A3 shows the exact number of “low” and “high” instances for arousal and valence per condition.

For an impression of which features vary with arousal and valence, and in what way, Table A4 displays the results of the Kruskal-Wallis tests, testing for differences between the “low” and “high” arousal and valence, for each of the features and separately per task. Out of 109 features, 59 show one or more significant effects. Effects are found in all categories, video- facial expression, gaze, head posture; and physiology- ECG and EDA. Across the various tasks, 42 features differentiated between low and high arousal levels. Of these, 32 (27 unique) are associated with camera data, while 10 are linked to physiological data. In contrast, for valence, 38 features are identified, with 14 originating from camera data and 24 (18 unique) from physiological data. Most effects are not consistent across tasks. The majority of features that differed between low and high arousal and valence levels were found within the the SSSTarousal.

### Modeling: specific-general context

Using logistic regression, we examined the possibility to distinguish between high and low self-reported arousal, and high and low self-reported valence on a single-trial level (data from one participant in one condition), in a specific context (models trained and tested within data originating from one condition), an intermediate context (combinations of conditions from the same setting), and a general context (including data from all conditions). In the first step, we used data from both the camera and physiological sensors (video-physio; first columns of Tables 1, 2).

**Table 1:** Accuracy for arousal models trained on physio-video, models trained on video data only, models trained on physiological data only, and expert performance for estimating arousal from various conditions as indicated in the left column, comprising specific contexts (green), intermediate contexts (yellow), and general context (red).

**Arousal—accuracy**				
**Condition**	**All features**	**Video features**	**Physiological features**	**Experts**
Baseline recording movie	*x*	*x*	*x*	*x*
Cognitive task: baseline	*x*	*x*	*x*	42%
Cognitive task: stress	**81%**	**72%**	52%	63%
Sing-a-song: baseline	**76%**	**65%**	**65%**	55%
Sing-a-song: stress	**84%**	**77%**	70%	46%
Public-speaking: baseline	62%	65%	69%	33%
Public-speaking: stress	**81%**	**75%**	49%	48%
Startle movie	**71%**	**68%**	**64%**	*x*
Cognitive task (baseline & stress)	**72%**	**67%**	**53%**	53%
Sing-a-song (baseline & stress)	**74%**	**67%**	**70%**	50%
Public-speaking (baseline & stress)	**64%**	**61%**	51%	41%
Cognitive and sing-a-song tasks	**69%**	**62%**	**65%**	51%
Cognitive, sing-a-song and public speaking tasks	**61%**	**58%**	**59%**	48%
Stress conditions from all tasks	**70%**	**68%**	55%	*x*
All data (all tasks including baseline recording)	**64%**	**61%**	**62%**	*x*

Crosses depict combinations for which no models could be created. Values above chance level are indicated in bold.

**Table 2:** Accuracy for valence models trained on physio-video, models trained on video data only, models trained on physiological data only, and expert performance for estimating arousal from various conditions as indicated in the left column, comprising specific contexts (green), intermediate contexts (yellow), and general context (red).

**Valence—accuracy**				
**Condition**	**All features**	**Video features**	**Physiological features**	**Experts**
Baseline recording movie	**82%**	57%	**71%**	*x*
Cognitive task: baseline	**70%**	**67%**	**66%**	49%
Cognitive task: stress	**78%**	**62%**	**73%**	47%
Sing-a-song: baseline	**71%**	**69%**	**66%**	50%
Sing-a-song: stress	**79%**	53%	**78%**	62%
Public-speaking: baseline	**71%**	**64%**	**66%**	64%
Public-speaking: stress	**74%**	**62%**	**64%**	64%
Startle movie	**70%**	58%	**70%**	*x*
Cognitive task (baseline & stress)	**65%**	**62%**	**60%**	48%
Sing-a-song (baseline & stress)	**74%**	**61%**	**66%**	56%
Public-speaking (baseline & stress)	**66%**	**68%**	**60%**	64%
Cognitive and sing-a-song tasks	**65%**	**60%**	**61%**	52%
Cognitive, sing-a-song and public speaking tasks	**60%**	**59%**	**56%**	**56%**
Stress conditions from all tasks	**63%**	**60%**	54%	*x*
All data (all tasks including baseline recording)	**60%**	**59%**	**55%**	*x*

Crosses depict combinations for which no models could be created. Values above chance level are indicated in bold.

**Figure 5:**
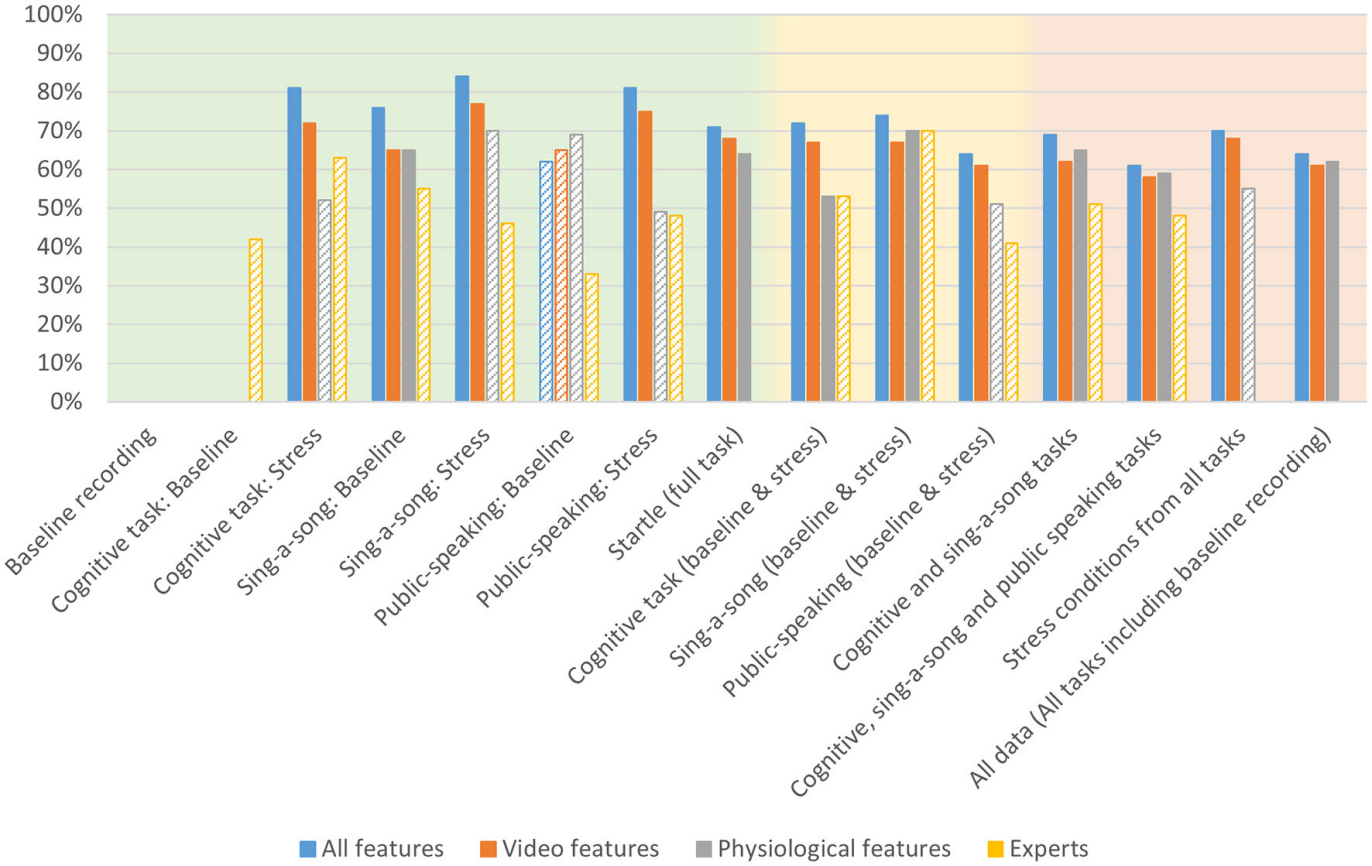
Accuracy for arousal models trained on all physio-video data, models trained on video data only, models trained on physiological data only and expert performance for estimating arousal from various conditions as indicated on the *x*-axis, comprising specific contexts (green background), intermediate contexts (yellow background), and general context (red background). Bars are missing for combinations for which no models could be created. Performance below chance level is indicated with a striped bar pattern.

Table 1 shows the modeling performance for classifying high and low arousal per condition and combination of conditions (see Figure 5 for a bar graph of data in Table 1). As mentioned in the previous section, models could not be created for the baseline recording condition and the baseline cognitive task given the low number of reported “high” arousal. As expected, context specific models (i.e., models trained and tested on data from one quite specific task) tended to produce better results, but all models performed higher than chance as indicated by the permutation tests, except for the context specific model “public-speaking: baseline”. Model performance ranged from 61% accuracy for the model combining the baseline and stress conditions of the three tasks, to 84% accuracy for the model on the SSST stress condition.

**Figure 6:**
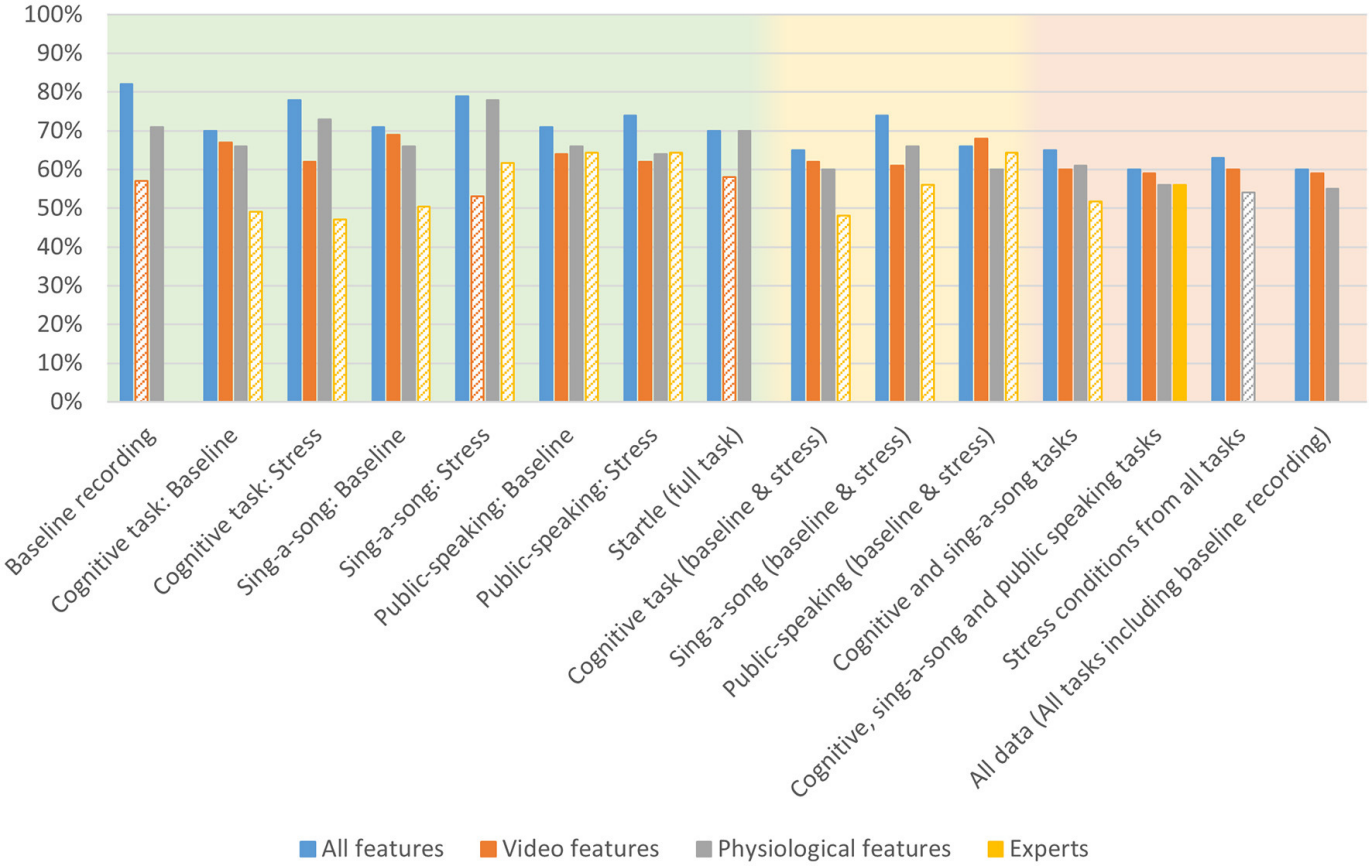
Accuracy for valence models trained on all physio-video data, models trained on video data only, models trained on physiological data only and expert performance for estimating arousal from various conditions as indicated on the x-axis, comprising specific contexts (green background), intermediate contexts (yellow background), and general context (red background). Bars are missing for combinations for which no models could be created. Performance below chance level is indicated with a striped bar pattern.

Table 2 shows modeling performance for classifying high and low valence (see Figure 6 for a bar graph of data in Table 2). Again, as expected, context specific models tended to produce better results, but all models performed higher than chance as indicated by the permutation tests. Performance ranged from 60% for all data, and baseline and stress conditions of the three tasks, to 82% for the baseline recording. Arousal models seem to perform slightly better than valence models.

Applying the models to the data of the five participants in the test set resulted in significant accuracies for the general models “cognitive and SSST tasks” (80% accuracy), “stress conditions from all tasks” (85%) and “all data” (68%). Other arousal models did not perform above chance. For valence, only the “stress conditions from all tasks” performed above chance (85%). Note that for the general context models we have more data points so that for these models, evaluation makes more sense.

### Modeling: modality

Tables 1, 2 present the accuracy results from the video-physio models (first columns) next to accuracy results of models that only used features from video data (second columns), or only from physiological data (third columns). In almost all context cases, video-physio models tend to perform best. Still, for general contexts, the advantage over video-only data is small.

The data do not suggest a clear advantage for either video features or physiological features. We see that in the general contexts (shaded in red) physiological models tend to perform better than video models in three out of four cases for arousal, while the opposite is true for valence. However, differences are small. In contrast to what we expected, we do not observe that physiological data have a special advantage over video features in general contexts. We also do not see an advantage for video features for valence, and an advantage for physiological features for arousal.

### Modeling: features

Exploring the features that were used in the different video-physio models indicates that the numbers of features used range between 3 and 30, where most models use 10 features or less. For arousal, 12 out of 13 models selected features of both video and physiological data; 10 out of 13 selected data from video, EDA and heart rate. For valence, 13 out of 15 models use data from video and physiological data; 11 out of 13 used data from video, EDA and heart rate. This suggests that each of the data sources provide complementary information. This is consistent with the advantage of the video-physio models as presented in the previous section. There are no specific features that stand out as particularly important for all conditions, neither for arousal, nor for valence, suggesting redundancy and context specificity. A complete overview of selected features per model is in Table A5 (arousal) and Table A6 (valence).

### Experts

The ratings as given by the three expert raters were divided into “low” and “high” valence and arousal just as the self-reported scores. To determine the interrater agreement between the different experts, Fleiss' Kappa scores were calculated. For arousal, the score was 0.27; for valence this was 0.24. Such values are regarded “fair” interrater agreement (Landis and Koch, 1977). The last columns in Tables 1, 2 show classification performance of experts for, respectively, arousal and valence. For none of the task-condition combinations, estimating arousal exceeded chance as determined by the permutation tests. For valence, performance only exceeded chance with 56% accuracy for the combined conditions from the three tasks. In all cases, the accuracy scores obtained by the models (left columns in Tables 1, 2) exceeded average expert scores, though the difference seems smaller for valence. Examining the results for the three experts individually did not change this pattern of results. The confusion matrices for each of the experts are shown in Table A7 (arousal) and Table A8 (valence).

## Discussion

In our study, participants performed various tasks (watching movies, public speaking, preparing to sing a song and cognitive tasks), aimed at eliciting varying levels of arousal and valence. Using models trained over participants, we showed that we could reliably estimate mental state using one to a few minutes' dynamic information from videos that captured their spontaneous behavior (facial action unit activity, head pose, and gaze) and physiological data (ECG and EDA). Up to 84% accuracy could be reached for classifying low vs. high arousal, and 82% for low vs. high valence. These values were reached using all features and staying within context, in these cases, respectively, preparing to sing a song and watching a neutral movie. Importantly, while performance dropped to 61–74% accuracy for arousal and 60–74% for valence, generalizing across contexts appeared to be possible by training models using data from combined contexts. This allows models to base themselves on features that behave relatively consistently across contexts. The multi-modal (video-physio) models tended to perform best, and indeed, almost all of these models are based on features from both video and physiological data, and usually features from both EDA and ECG. This is the case even though the number of used features is not exceptionally high (most models using 3–10 features). This indicates that the different modalities contain complementary information. Still, and good news for applied settings, the drop in performance when only using video data is modest. The arguably most difficult, but for some applications most valuable case, evaluating mental state across all conditions based on video data only, reaches an accuracy of about 60% for both arousal and valence, which is well-above chance.

The mentioned accuracies are based on the validation sets. Performance of the models on a completely independent testing set, consisting of data of five participants, reached above chance accuracies for some general context models. While results on an independent test set are the gold standard of evaluating models, given scarcity of data it is common in this type of research to only base results on validation sets. Our test set of five participants is not enough to make for a representative sample, but it prevented reducing the validation sets further, and it gives a very first impression of the generalizability of our models to completely new participants. Our finding that accuracies for models applied to the test set seem to be better when task conditions are combined compared to when not hint at an increasing robustness of models trained using a large variation of data.

Our models estimate self-reported arousal and valence better than human expert observers do, also models that do not take into account information that humans do not have access to (namely, physiological information) and are based only on visual (video) information. In fact, in our study, humans did not perform better than chance in all but one context cases.

One may argue that expert observers might estimate individuals' mental state better than individuals do themselves, and that that is the reason of the mismatch. We tried to gain support for this possibility by correlating individuals' scores on traits (perceived stress as obtained from the PSS; resilience scale, neuroticism) to the individuals' average self-reported arousal score on the six task conditions; and correlating these trait scores to the average expert-judged arousal score on the six task conditions. The premise is that if experts judge individuals better on their momentary experienced arousal than the individuals themselves, they should show more or better relations to trait as obtained from validated questionnaire tools. However, results showed no correlations between trait scores and expert-judgements, whereas there was a positive, significant association between PSS score and average self-reported arousal.

Another possible reason for the mismatch is that experts based their judgement on less data (10 s per condition), and did not have access to an example of distribution VAS scores. On the other hand, experts had knowledge that the model did not have, namely knowledge of (the meaning of) the task at hand, knowledge of the meaning of VAS scores and knowledge on the identity of the participants (i.e., they could compare responses within participants). In sum, models and humans have different information to base their judgement on, which may have contributed to the mismatch. This is hard or impossible to equalize. It is also important to realize that in almost all real life situations, selection psychologists would not judge mental state from facial expression in a way that is comparable to the study. They would communicate with individuals and check how they respond to certain questions. Still, our rough comparison between performance of humans and models indicates that the models' performance is not trivial, and that models may be of added value to expert judgement, especially in innovative, automized applicant screening methods, or very different applications where we would want to complement and/or reduce effort of human expert observers.

Our study provided positive results for monitoring mental state (arousal and valence) across individuals and across tasks using video measures in desktop situations. This may be helpful in selection procedures, where individuals are prone to show reporting bias, and where one may wish to examine whether an individual scores low on arousal and high in valence during a certain, potentially stressful task. This might be one of the tasks described in this study, or a certain serious gaming task. Similar models may also be used for adapting the stress level in a serious gaming task to the individual so that individuals are assessed, or trainees trained, while performing a certain task under similarly high levels of stress. This would allow comparing performance under stress between individuals. Another application may be in specific forms of biofeedback, e.g., adapting anxiety-evoking stimuli in virtual reality to appropriate levels in exposure therapy (Repetto et al., 2009; Brouwer et al., 2011; Rahman et al., 2023). While our study showed that we can generalize models across tasks by training models using data from different tasks, it is advisable to, whenever possible, stay within context, since this will result in better performance. This would be possible for certain applications. In addition, context (e.g., a certain element of a serious game that is being performed) may simply be identified using knowledge of the content of the user's display or task at hand, and used to apply the proper model.

In this study, we focused on self-rated arousal and valence, anticipating that arousal might be better captured by physiology, and valence by video data. Also, we expected that physiology may be relatively valuable for general context models. Results did not support these expectations. At least under the circumstances tested, information on both arousal and valence can be quite well-captured using video data only.

While a state of (undesirable) stress can be operationalized by low valence and high arousal, future studies may focus on a single item, arguably more intuitive measure of self-reported stress. This may improve self-reported labels and therewith improve model results. Depending on the application in mind, future analyses and future studies can study more or continuous estimates rather than discrete high and low categorization. Most importantly, for better validation of the models, and better estimate of what they can do in applications, we need to collect more data and include validation with independent test sets.

However, accurate a model performs in estimating the mental state of an individual, in terms of matching self-report or even in other terms, for applications the ultimate question remains whether exploiting these estimates in one way or the other improves individuals' or organizational performance and wellbeing. Such longitudinal research, comparing methods that involve automatic mental state monitoring to current practice, is important to definitely show the added value of mental state monitoring using video and physiological data.

## Data availability statement

The raw data supporting the conclusions of this article will be made available by the authors, without undue reservation.

## Ethics statement

The studies involving humans were approved by Internal Review Board at TNO (reference number 2022-093). The studies were conducted in accordance with the local legislation and institutional requirements. The participants provided their written informed consent to participate in this study.

## Author contributions

JB: Conceptualization, Data curation, Formal analysis, Investigation, Methodology, Software, Writing – original draft, Writing – review & editing. IS: Conceptualization, Data curation, Formal analysis, Investigation, Methodology, Software, Supervision, Writing – review & editing. PP: Writing – original draft, Writing – review & editing. KH: Conceptualization, Writing – review & editing. MN: Conceptualization, Writing – review & editing. WK: Conceptualization, Funding acquisition, Project administration, Writing – review & editing. A-MB: Conceptualization, Methodology, Supervision, Writing – original draft, Writing – review & editing.
