# Supplementary material for: Detection of arousal and valence from facial expressions and physiological responses evoked by different types of stressors
Source: Front Neuroergon. 2024 Mar 15;5:1338243. doi: 10.3389/fnrgo.2024.1338243 (PMC10978716; doi:10.3389/fnrgo.2024.1338243)
Supplement: Supplementary file 1 [file Data_Sheet_1.docx]

# Appendix

**Table A1**: Complete overview of extracted video features. For action units (AU), the mean intensity was calculated.

|  |  | Extracted features | | |
| --- | --- | --- | --- | --- |
| Measure | Description | Mean | Variance over time | Degree of presence |
| AU01 | inner brow raiser | x | x | x |
| AU02 | outer brow raiser | x | x | x |
| AU04 | brow lowerer | x | x | x |
| AU06 | cheek raiser | x | x | x |
| AU07 | lid tightener | x | x | x |
| AU09 | nose wrinkler | x | x | x |
| AU10 | upper lip raiser | x | x | x |
| AU12 | lip corner puller | x | x | x |
| AU14 | dimpler | x | x | x |
| AU15 | lip corner depressor | x | x | x |
| AU17 | chin raiser | x | x | x |
| AU20 | lip stretcher | x | x | x |
| AU23 | lip tightener | x | x | x |
| AU25 | lips part | x | x | x |
| AU26 | jaw drop | x | x | x |
| AU28 | lip suck |  |  | x |
| AU45 | blink | x | x | x |
| GazeX | horizontal gaze | x | x |  |
| GazeY | vertical gaze | x | x |  |
| PoseTx | horizontal position of head | x | x |  |
| PoseTy | vertical position of head | x | x |  |
| PoseTz | distance from camera | x | x |  |
| PoseRx | pitch rotation | x | x |  |
| PoseRy | yaw rotation | x | x |  |
| PoseRz | roll rotation | x | x |  |

**Table A2**: Complete overview of initially extracted physiological features from EDA and ECG signals.

|  |  |  | Extracted features | | | | | | |  |
| --- | --- | --- | --- | --- | --- | --- | --- | --- | --- | --- |
| Sensortype | Measure | Mean | Standard deviation | Minimal amplitude | Median amplitude | Maximum amplitude | Area under the curve | Kurtosis | Skewness | Other |
| EDA | EDA | x | x | x | x | x | x | x | x |  |
| EDA | SCR | x | x | x | x | x | x | x | x |  |
| EDA | average phasic driver |  |  |  |  |  |  |  |  | x |
| EDA | mean tonic activity |  |  |  |  |  |  |  |  | x |
| EDA | number of significant SCRs |  |  |  |  |  |  |  |  | x |
| EDA | Sum of significant SCR amplitudes |  |  |  |  |  |  |  |  | x |
| EDA | maximum phasic activity |  |  |  |  |  |  |  |  | x |
| EDA | area of phasic driver |  |  |  |  |  |  |  |  | x |
| EDA | response latency of first significant SCR |  |  |  |  |  |  |  |  | x |
| ECG | HR | x | x | x | x | x | x | x | x |  |
| ECG | VLF |  |  |  |  |  |  |  |  | x |
| ECG | LF |  |  |  |  |  |  |  |  | x |
| ECG | HF |  |  |  |  |  |  |  |  | x |
| ECG | LF/HF |  |  |  |  |  |  |  |  | x |
| ECG | RMSSD |  |  |  |  |  |  |  |  | x |
| ECG | IBI | x | x | x | x | x | x | x | x |  |

**Table A3**: Absolute numbers of ‘low’ and ‘high’ responses for rated arousal and valence per condition.

|  | Arousal | | Valence | |
| --- | --- | --- | --- | --- |
|  | N low | N high | N low | N high |
| Baseline recording | 48 | 2 | 12 | 38 |
| Cognitive task: Baseline | 45 | 4 | 21 | 28 |
| Cognitive task: Stress | 16 | 33 | 22 | 27 |
| Sing-a-song: Baseline | 36 | 13 | 22 | 27 |
| Sing-a-song: Stress | 11 | 37 | 38 | 10 |
| Public-speaking: Baseline | 39 | 10 | 13 | 36 |
| Public-speaking: Stress | 24 | 25 | 23 | 26 |
| Startle | 22 | 27 | 21 | 28 |

**Table A4**: Results of the Kruskal-Wallis tests, testing for differences between the ‘low’ and ‘high’ arousal (left columns) and valence (right columns), for each of the features and separately per task. *, ** and *** indicate significance levels of 0.05, 0.01 and 0.001 respectively. Only features with one or more significant effects are included in the table. For significant effects, the direction is indicated where an upward pointing arrow means higher feature values for high arousal or valence than for low arousal and valence, and a downward pointing arrow meaning the opposite. The arrow pointing to the left refers to a location more to the left during high compared to low arousal. Features are coded by color, indicating facial expression (green), gaze (blue), head pose (red), ECG features (yellow) and EDA features (gray).

|  |  |  | **Arousal** |  |  |  | **Valence** |  |  |
| --- | --- | --- | --- | --- | --- | --- | --- | --- | --- |
| **Feature** | Feature definition | Cognitive task | SSST | Speaking task | Direction of effect | Cognitive task | SSST | Speaking task | Direction of effect |
| pAU01 | Inner brow raiser (mean presence) |  |  | * | v |  |  |  |  |
| mAU02 | Outer brow raiser (mean intensity) |  |  |  |  |  |  | * | ^ |
| pAU02 | Outer brow raiser (mean presence) |  |  | ** | v |  |  |  |  |
| mAU04 | Brow lowerer (mean intensity) |  |  |  |  |  | ** |  | ^ |
| sdAU04 | Brow lowerer (variance intensity) |  |  |  | ^ |  |  |  |  |
| mAU06 | Cheek raiser (mean intensity) |  | * |  | ^ |  |  |  |  |
| pAU06 | Cheek raiser (mean presence) |  | * |  | ^ |  |  | * | ^ |
| sdAU06 | Cheek raiser (variance intensity) |  | * |  | ^ |  |  |  |  |
| pAU07 | Lid tightener (mean presence) |  | * |  | ^ |  | * |  | v |
| pAU09 | Nose wrinkler (mean presence) |  | ** |  | ^ |  |  |  |  |
| mAU10 | Upper lip raiser (mean intensity) |  | *** | * | ^ |  |  |  |  |
| pAU10 | Upper lip raiser (mean presence) | * | *** | * | ^ |  |  |  |  |
| sdAU10 | Upper lip raiser (variance intensity) |  | *** |  | ^ |  |  |  |  |
| mAU12 | Lip corner puller (mean intensity) |  | ** |  | ^ | * |  |  | v |
| pAU12 | Lip corner puller (mean presence) |  | *** |  | ^ |  |  |  |  |
| sdAU12 | Lip corner puller (variance intensity) |  | *** |  | ^ | * |  |  | v |
| mAU14 | Dimpler (mean intensity) |  | ** |  | v |  |  |  |  |
| sdAU14 | Dimpler (variance intenstiy) |  | *** |  | ^ |  |  |  |  |
| mAU15 | Lip corner depressor (mean intensity) |  | * |  | v |  |  |  |  |
| mAU17 | Chin raiser (mean intensity) |  |  |  |  |  | * |  | ^ |
| mAU25 | Lips part (mean intensity) |  | ** |  | ^ |  | * |  | v |
| pAU25 | Lips part (mean presence) |  | ** |  | ^ |  |  |  |  |
| sdAU25 | Lips part (variance intensity) | * | *** |  | ^ |  |  | * | ^ |
| mAU26 | Jaw drop (mean intensity) |  | ** |  | ^ |  |  |  |  |
| pAU26 | Jaw drop (mean presence) |  | ** | * | ^/v |  |  |  |  |
| sdAU26 | Jaw drop (variance intensity) |  | * |  | ^ |  |  |  |  |
| mAU45 | Blink (mean intensity) | * |  |  | v |  |  |  |  |
| pAU45 | Blink (mean presence) | * |  |  | ^ |  |  | * | ^ |
| sdAU45 | Blink (variance intensity) | * |  |  | v |  |  |  |  |
| mGazeX | Mean gaze-angle in x-direction |  |  |  |  |  |  | * | v |
| sdGazeY | Variance of gaze-angle in y-direction |  |  |  |  |  |  | ** | ^ |
| mPoseTx | Mean location of head on x-axis | * |  |  | < |  |  |  |  |
| sdPoseTx | Variance in location of head on x-axis |  |  |  |  |  | * |  | v |
| sdPoseTy | Variance in location of head on y-axis | *** |  |  | ^ |  |  |  |  |
| sdPoseTz | Variance in distance from camera |  |  |  |  | * |  |  | v |
| LF HRV | Power in low frequency band of HRV |  |  |  |  |  |  | ** | ^ |
| RMSSD | Root mean squared of succesive differences in HRV |  |  |  |  | * |  |  | v |
| min HR | Minimum heart rate |  |  |  |  |  |  | * | v |
| max HR | Maximum heart rate |  |  |  |  | ** | ** |  | v |
| HR | Average heart rate |  |  |  |  |  | * |  | v |
| sd HR | Standard deviation of heart rate |  | * |  | ^ | * | ** |  | v |
| skew HR | Skewness of heart rate |  |  |  |  |  | * |  | ^ |
| AUC HR | Area under the curve of heart rate |  |  |  |  | * |  |  | v |
| EDA | Mean electrodermal activity |  |  |  |  | ** | * |  | v |
| sd EDA | Standard deviation of electrodermal activity |  | ** |  | ^ |  |  |  |  |
| maxEDA | Maximum electrodermal activity |  |  |  |  | * | * |  | v |
| minEDA | Minimum electrodermal activity |  |  |  |  | * |  |  | v |
| ampEDA | Electrodermal activity amplitude |  | * |  | ^ |  |  |  |  |
| mEDA | Median electrodermal activity |  |  |  |  | ** | * |  | v |
| AUC EDA | Area under the curve of electrodermal activity |  |  |  |  | ** |  |  | v |
| skew EDA | Skewness of electrodermal activity |  | ** |  | v |  |  |  |  |
| SCR | Mean skin conductance response |  | ** |  | ^ |  | * |  | v |
| SCL | Mean skin conductance level |  |  |  |  | ** | * |  | v |
| sd SCR | Standard deviation of skin conductance response |  | * |  | ^ |  |  |  |  |
| AUC SCR | Area under the curve of skin conductance response |  | * |  | ^ |  |  |  |  |
| nSCR | Mean non-specific skin conductance response |  | * |  | ^ |  | * |  | v |
| mSCR | Median skin conductance response |  | * |  | ^ |  | * |  | v |
| kur SCR | Kurtosis skin conductance response |  |  |  |  |  | * |  | ^ |
| maxSCR | Maximum skin conductance response |  | * |  | ^ |  |  |  |  |

**Table A5**: Selected features (rows) per arousal model (columns) indicated by a filled cell. Features are ranked according to the number of times they were selected. Models are coded by color, indicating specific context models (green), intermediate context models (yellow) and general context models (red).

| **Predicting Arousal** |  |  |  |  |  |  |  |  |  |  |  |  |  |  |  |  |
| --- | --- | --- | --- | --- | --- | --- | --- | --- | --- | --- | --- | --- | --- | --- | --- | --- |
| **Feature** | **Cognitive task: Stress** | **Cognitive task: Baseline** | **Sing-a-song: Baseline** | **Sing-a-song: Stress** | **Public-speaking: Stress** | **Public-speaking: Baseline** | **Startle (full)** | **Baseline recording** | **Cognitive task (baseline & stress)** | **Sing-a-song (baseline & stress)** | **Public-speaking (baseline & stress)** | **Cognitive and sing-a-song tasks** | **Cognitive, sing-a-song and public speaking** | **Stress conditions from all tasks** | **All data (All tasks including baseline recording)** | **Count (excl baseline and cognitive baseline)** |
| Upper lip raiser (mean intensity) | 0 | 0 | 1 | 1 | 1 | 0 | 1 | 0 | 0 | 1 | 1 | 1 | 1 | 1 | 1 | 10 |
| Outer brow raiser (mean presence) | 1 | 0 | 0 | 0 | 1 | 0 | 1 | 0 | 0 | 0 | 1 | 0 | 1 | 1 | 1 | 7 |
| Mean location of head on x-axis | 1 | 0 | 0 | 0 | 0 | 0 | 0 | 0 | 1 | 0 | 0 | 1 | 1 | 1 | 1 | 6 |
| Variance of gaze-angle in x-direction | 0 | 0 | 0 | 0 | 0 | 0 | 1 | 0 | 0 | 0 | 0 | 1 | 1 | 1 | 1 | 5 |
| Average phasic driver component of EDA signal | 0 | 1 | 0 | 0 | 0 | 1 | 0 | 0 | 0 | 0 | 0 | 1 | 1 | 0 | 1 | 4 |
| Cheek raiser (mean presence) | 0 | 0 | 0 | 0 | 0 | 1 | 0 | 0 | 0 | 0 | 0 | 1 | 1 | 0 | 1 | 4 |
| Lip corner puller (variance intensity) | 1 | 0 | 0 | 1 | 0 | 0 | 0 | 0 | 0 | 0 | 0 | 1 | 0 | 1 | 0 | 4 |
| Area under the curve of heart rate signal | 0 | 0 | 0 | 0 | 0 | 0 | 0 | 1 | 0 | 0 | 0 | 1 | 0 | 1 | 1 | 3 |
| Lip corner depressor (mean intensity) | 1 | 0 | 0 | 0 | 0 | 0 | 0 | 0 | 0 | 0 | 0 | 1 | 0 | 1 | 0 | 3 |
| Dimpler (mean intensity | 0 | 0 | 0 | 1 | 0 | 0 | 0 | 0 | 0 | 0 | 0 | 1 | 0 | 1 | 0 | 3 |
| Lid tightener (mean intensity) | 0 | 1 | 0 | 0 | 1 | 0 | 0 | 0 | 0 | 0 | 1 | 0 | 0 | 1 | 0 | 3 |
| Standard deviation in skin conductance response | 1 | 0 | 0 | 0 | 1 | 0 | 0 | 0 | 0 | 0 | 0 | 0 | 0 | 1 | 0 | 3 |
| Inner brow raiser (variance intensity) | 0 | 0 | 0 | 1 | 1 | 0 | 0 | 0 | 0 | 0 | 0 | 0 | 0 | 1 | 0 | 3 |
| Upper lip raiser (mean presence) | 0 | 0 | 0 | 0 | 1 | 0 | 0 | 0 | 0 | 0 | 1 | 0 | 0 | 1 | 0 | 3 |
| Skewness of interbeat interval | 0 | 0 | 0 | 1 | 0 | 0 | 0 | 0 | 0 | 0 | 0 | 1 | 1 | 0 | 0 | 3 |
| Brow lowerer (mean presence) | 1 | 1 | 0 | 1 | 0 | 1 | 0 | 1 | 0 | 0 | 0 | 0 | 0 | 0 | 0 | 3 |
| Kurtosis of heart rate signal | 0 | 0 | 0 | 0 | 0 | 0 | 0 | 0 | 0 | 0 | 0 | 1 | 0 | 0 | 1 | 2 |
| Mean yaw head rotation | 1 | 0 | 0 | 0 | 0 | 0 | 0 | 0 | 0 | 0 | 0 | 0 | 0 | 0 | 1 | 2 |
| Skewness of EDA signal | 0 | 0 | 0 | 1 | 0 | 0 | 0 | 0 | 0 | 0 | 0 | 0 | 0 | 0 | 1 | 2 |
| Lip corner depressor (variance intensity) | 1 | 0 | 0 | 0 | 0 | 0 | 0 | 0 | 0 | 0 | 0 | 0 | 0 | 1 | 0 | 2 |
| Skewness of EDA signal | 0 | 0 | 0 | 0 | 0 | 0 | 0 | 0 | 0 | 1 | 0 | 0 | 0 | 1 | 0 | 2 |
| Minimum amplitude of skin conductance response | 0 | 0 | 0 | 0 | 0 | 0 | 0 | 0 | 0 | 0 | 1 | 0 | 0 | 1 | 0 | 2 |
| Area under the curve of EDA signal | 0 | 1 | 0 | 0 | 0 | 0 | 0 | 0 | 0 | 0 | 0 | 1 | 1 | 0 | 0 | 2 |
| Lid tightener (mean presence) | 0 | 0 | 0 | 1 | 0 | 0 | 0 | 1 | 0 | 0 | 0 | 1 | 0 | 0 | 0 | 2 |
| Root mean squared of successive differences in HRV | 0 | 0 | 1 | 0 | 0 | 0 | 0 | 0 | 0 | 0 | 0 | 1 | 0 | 0 | 0 | 2 |
| Kurtosis of skin conductance response | 0 | 0 | 0 | 1 | 0 | 0 | 0 | 0 | 0 | 0 | 0 | 1 | 0 | 0 | 0 | 2 |
| Nose wrinkler (variance intensity) | 0 | 0 | 0 | 1 | 0 | 0 | 0 | 0 | 0 | 0 | 0 | 1 | 0 | 0 | 0 | 2 |
| Cheek raiser (variance intensity) | 0 | 0 | 0 | 1 | 0 | 0 | 0 | 0 | 0 | 0 | 0 | 1 | 0 | 0 | 0 | 2 |
| Dimpler (variance intensity) | 0 | 0 | 0 | 1 | 0 | 0 | 0 | 0 | 0 | 0 | 0 | 1 | 0 | 0 | 0 | 2 |
| Upper lip raiser (variance intensity) | 0 | 0 | 0 | 1 | 0 | 0 | 0 | 0 | 0 | 0 | 0 | 1 | 0 | 0 | 0 | 2 |
| Standard deviation of interbeat interval | 0 | 0 | 0 | 0 | 0 | 0 | 0 | 0 | 0 | 1 | 0 | 1 | 0 | 0 | 0 | 2 |
| Mean gaze-angle in x-direction | 1 | 0 | 0 | 0 | 0 | 0 | 1 | 1 | 0 | 0 | 0 | 0 | 0 | 0 | 0 | 2 |
| Mean pitch rotation | 1 | 0 | 0 | 0 | 0 | 0 | 0 | 1 | 1 | 0 | 0 | 0 | 0 | 0 | 0 | 2 |
| Mean location of head on y-axis | 0 | 0 | 0 | 1 | 0 | 0 | 0 | 1 | 0 | 0 | 1 | 0 | 0 | 0 | 0 | 2 |
| Blink (mean intensity) | 1 | 0 | 0 | 0 | 0 | 0 | 0 | 0 | 1 | 0 | 0 | 0 | 0 | 0 | 0 | 2 |
| Variance in location of head on y-axis | 1 | 0 | 0 | 0 | 1 | 0 | 0 | 0 | 0 | 0 | 0 | 0 | 0 | 0 | 0 | 2 |
| Outer brow raiser (variance intensity) | 1 | 0 | 0 | 1 | 0 | 0 | 0 | 0 | 0 | 0 | 0 | 0 | 0 | 0 | 0 | 2 |
| Standard deviation in distance from camera | 0 | 0 | 0 | 1 | 1 | 0 | 0 | 0 | 0 | 0 | 0 | 0 | 0 | 0 | 0 | 2 |
| Jaw drop (mean presence) | 0 | 0 | 0 | 0 | 0 | 1 | 0 | 0 | 0 | 0 | 1 | 0 | 0 | 0 | 0 | 2 |
| Lip tightener (mean presence) | 0 | 1 | 0 | 0 | 0 | 0 | 0 | 0 | 0 | 0 | 0 | 0 | 0 | 1 | 0 | 1 |
| Area under the curve of IBI | 0 | 1 | 0 | 0 | 0 | 0 | 0 | 0 | 0 | 0 | 0 | 0 | 0 | 1 | 0 | 1 |
| Variance in distance from camera | 0 | 0 | 0 | 0 | 0 | 0 | 0 | 0 | 0 | 0 | 0 | 0 | 0 | 1 | 0 | 1 |
| Mean roll rotation | 0 | 0 | 0 | 0 | 0 | 0 | 0 | 0 | 0 | 0 | 0 | 0 | 0 | 1 | 0 | 1 |
| Inner brow raiser (mean presence)) | 0 | 0 | 0 | 0 | 0 | 0 | 0 | 0 | 0 | 0 | 0 | 0 | 0 | 1 | 0 | 1 |
| Standard deviation in interbeat interval | 0 | 0 | 0 | 0 | 0 | 0 | 0 | 0 | 0 | 0 | 0 | 0 | 1 | 0 | 0 | 1 |
| Dimpler (mean intensity) | 0 | 0 | 0 | 0 | 0 | 0 | 0 | 0 | 0 | 0 | 0 | 0 | 1 | 0 | 0 | 1 |
| Standard deviation in skin conductance component | 0 | 0 | 0 | 0 | 0 | 0 | 0 | 1 | 0 | 0 | 0 | 1 | 0 | 0 | 0 | 1 |
| Minimum of EDA signal | 0 | 1 | 0 | 0 | 0 | 0 | 0 | 0 | 0 | 0 | 0 | 1 | 0 | 0 | 0 | 1 |
| Skewness of the EDA signal | 0 | 0 | 0 | 0 | 0 | 0 | 0 | 0 | 0 | 0 | 0 | 1 | 0 | 0 | 0 | 1 |
| Lip corner puller (mean presence) | 0 | 0 | 0 | 0 | 0 | 0 | 0 | 0 | 0 | 0 | 0 | 1 | 0 | 0 | 0 | 1 |
| Lips part (variance intensity) | 0 | 0 | 0 | 0 | 0 | 0 | 0 | 0 | 0 | 0 | 0 | 1 | 0 | 0 | 0 | 1 |
| Skewness of heart rate signal | 0 | 0 | 0 | 0 | 0 | 0 | 0 | 0 | 0 | 0 | 0 | 1 | 0 | 0 | 0 | 1 |
| Mean skin conductance component | 0 | 0 | 0 | 0 | 0 | 0 | 0 | 0 | 0 | 0 | 0 | 1 | 0 | 0 | 0 | 1 |
| Lips part (mean intensity) | 0 | 0 | 0 | 0 | 0 | 0 | 0 | 0 | 0 | 0 | 0 | 1 | 0 | 0 | 0 | 1 |
| Skewness of skin conductance response | 0 | 0 | 0 | 0 | 0 | 0 | 0 | 0 | 0 | 0 | 0 | 1 | 0 | 0 | 0 | 1 |
| Median amplitude of skin conductance response | 0 | 0 | 0 | 0 | 0 | 0 | 0 | 0 | 0 | 0 | 0 | 1 | 0 | 0 | 0 | 1 |
| Brow lowerer (mean intensity) | 1 | 1 | 0 | 0 | 0 | 0 | 0 | 1 | 0 | 0 | 0 | 0 | 0 | 0 | 0 | 1 |
| Mean gaze-angle in y-direction | 1 | 0 | 0 | 0 | 0 | 0 | 0 | 1 | 0 | 0 | 0 | 0 | 0 | 0 | 0 | 1 |
| Median heart rate | 0 | 0 | 0 | 1 | 0 | 0 | 0 | 1 | 0 | 0 | 0 | 0 | 0 | 0 | 0 | 1 |
| Maximum heart rate | 0 | 0 | 0 | 0 | 0 | 1 | 0 | 1 | 0 | 0 | 0 | 0 | 0 | 0 | 0 | 1 |
| Number of significant SCRs within response window | 0 | 1 | 0 | 0 | 0 | 0 | 0 | 0 | 0 | 0 | 1 | 0 | 0 | 0 | 0 | 1 |
| Minimal skin conductance component | 1 | 0 | 0 | 0 | 0 | 0 | 0 | 0 | 0 | 0 | 0 | 0 | 0 | 0 | 0 | 1 |
| Lip stretcher (variance intensity) | 1 | 0 | 0 | 0 | 0 | 0 | 0 | 0 | 0 | 0 | 0 | 0 | 0 | 0 | 0 | 1 |
| Lip stretcher (mean intensity) | 1 | 0 | 0 | 0 | 0 | 0 | 0 | 0 | 0 | 0 | 0 | 0 | 0 | 0 | 0 | 1 |
| Kurtosis of EDA signal | 1 | 0 | 0 | 0 | 0 | 0 | 0 | 0 | 0 | 0 | 0 | 0 | 0 | 0 | 0 | 1 |
| Blink (variance intensity) | 1 | 0 | 0 | 0 | 0 | 0 | 0 | 0 | 0 | 0 | 0 | 0 | 0 | 0 | 0 | 1 |
| Lip suck (mean presence) | 0 | 0 | 1 | 0 | 0 | 0 | 0 | 0 | 0 | 0 | 0 | 0 | 0 | 0 | 0 | 1 |
| Outer brow raiser (mean intensity) | 0 | 0 | 0 | 1 | 0 | 0 | 0 | 0 | 0 | 0 | 0 | 0 | 0 | 0 | 0 | 1 |
| Response latency of first significant SCR | 0 | 0 | 0 | 1 | 0 | 0 | 0 | 0 | 0 | 0 | 0 | 0 | 0 | 0 | 0 | 1 |
| Lip corner puller (mean intensity) | 0 | 0 | 0 | 1 | 0 | 0 | 0 | 0 | 0 | 0 | 0 | 0 | 0 | 0 | 0 | 1 |
| Minimum of skin conductance response | 0 | 0 | 0 | 0 | 1 | 0 | 0 | 0 | 0 | 0 | 0 | 0 | 0 | 0 | 0 | 1 |
| Maximum value of phasic activity | 0 | 0 | 0 | 0 | 1 | 0 | 0 | 0 | 0 | 0 | 0 | 0 | 0 | 0 | 0 | 1 |
| Power in high frequency band of HRV | 0 | 0 | 0 | 0 | 0 | 0 | 1 | 0 | 0 | 0 | 0 | 0 | 0 | 0 | 0 | 1 |
| Variance of gaze-angle in y-direction | 0 | 0 | 0 | 0 | 0 | 0 | 0 | 0 | 0 | 0 | 1 | 0 | 0 | 0 | 0 | 1 |
| Ratio between power in high and low frequency bands of HRV | 0 | 0 | 0 | 0 | 0 | 0 | 0 | 0 | 0 | 0 | 1 | 0 | 0 | 0 | 0 | 1 |

**Table A6**: Selected features (rows) per valence model (columns) indicated by a filled cell. Features are ranked according to the number of times they were selected. Models are coded by color, indicating specific context models (green), intermediate context models (yellow) and general context models (red).

| **Predicting Valence** |  |  |  |  |  |  |  |  |  |  |  |  |  |  |  |  |
| --- | --- | --- | --- | --- | --- | --- | --- | --- | --- | --- | --- | --- | --- | --- | --- | --- |
| **Feature** | **Cognitive task: Stress** | **Cognitive task: Baseline** | **Sing-a-song: Baseline** | **Sing-a-song: Stress** | **Public-speaking: Stress** | **Public-speaking: Baseline** | **Startle (full)** | **Baseline recording** | **Cognitive task (baseline & stress)** | **Sing-a-song (baseline & stress)** | **Public-speaking (baseline & stress)** | **Cognitive and sing-a-song tasks** | **Cognitive, sing-a-song and public speaking** | **Stress conditions from all tasks** | **All data (All tasks including baseline recording)** | **Count** |
| Standard deviation in distance from camera | 0 | 0 | 1 | 1 | 0 | 0 | 0 | 0 | 0 | 1 | 0 | 1 | 1 | 0 | 0 | 5 |
| Maximum interbeat interval | 0 | 0 | 0 | 0 | 1 | 1 | 0 | 0 | 0 | 0 | 1 | 0 | 0 | 1 | 0 | 4 |
| Power in high frequency band of HRV | 1 | 1 | 1 | 0 | 0 | 0 | 1 | 0 | 0 | 0 | 0 | 0 | 0 | 0 | 0 | 4 |
| Inner brow raiser (mean intensity) | 0 | 0 | 1 | 0 | 0 | 0 | 1 | 0 | 0 | 1 | 0 | 1 | 0 | 0 | 0 | 4 |
| Skewness of skin conductance response | 0 | 0 | 1 | 0 | 1 | 1 | 0 | 0 | 0 | 0 | 1 | 0 | 0 | 0 | 0 | 4 |
| Maximum heart rate | 0 | 0 | 1 | 0 | 0 | 0 | 0 | 0 | 0 | 1 | 0 | 1 | 1 | 0 | 0 | 4 |
| Area under the curve of the interbeat intervals | 0 | 0 | 0 | 0 | 0 | 0 | 0 | 0 | 0 | 0 | 0 | 0 | 1 | 1 | 1 | 3 |
| Lid tightener (variance intensity) | 0 | 0 | 0 | 0 | 0 | 0 | 0 | 1 | 0 | 0 | 0 | 0 | 1 | 0 | 1 | 3 |
| Standard deviation in heart rate | 0 | 0 | 1 | 0 | 0 | 0 | 0 | 0 | 0 | 1 | 0 | 1 | 0 | 0 | 0 | 3 |
| Lip stretcher (variance intensity) | 0 | 0 | 0 | 1 | 0 | 0 | 0 | 1 | 0 | 1 | 0 | 0 | 0 | 0 | 0 | 3 |
| Lip corner puller (mean presence) | 0 | 0 | 0 | 0 | 1 | 0 | 0 | 0 | 0 | 0 | 1 | 0 | 1 | 0 | 0 | 3 |
| Brow lowerer (mean intensity) | 0 | 0 | 0 | 0 | 0 | 0 | 0 | 1 | 0 | 1 | 0 | 1 | 0 | 0 | 0 | 3 |
| Skewness in heart rate signal | 0 | 0 | 0 | 0 | 0 | 0 | 0 | 1 | 0 | 1 | 0 | 1 | 0 | 0 | 0 | 3 |
| Minimum of EDA signal | 0 | 0 | 0 | 0 | 0 | 0 | 0 | 0 | 0 | 0 | 0 | 1 | 0 | 0 | 1 | 2 |
| Response latency of first significant SCR | 0 | 0 | 1 | 0 | 0 | 0 | 0 | 0 | 0 | 0 | 0 | 0 | 0 | 1 | 0 | 2 |
| Standard deviation in pitch rotation | 0 | 0 | 0 | 0 | 1 | 0 | 0 | 0 | 0 | 0 | 0 | 0 | 0 | 1 | 0 | 2 |
| Kurtosis of EDA signal | 0 | 0 | 0 | 0 | 0 | 0 | 0 | 0 | 0 | 0 | 0 | 0 | 1 | 1 | 0 | 2 |
| Variance in roll head rotation | 1 | 0 | 0 | 0 | 0 | 0 | 0 | 0 | 1 | 0 | 0 | 0 | 0 | 0 | 0 | 2 |
| Standard deviation in skin conductance response | 1 | 0 | 0 | 0 | 0 | 0 | 0 | 0 | 0 | 0 | 0 | 1 | 0 | 0 | 0 | 2 |
| Minimum interbeat interval | 1 | 0 | 1 | 0 | 0 | 0 | 0 | 0 | 0 | 0 | 0 | 0 | 0 | 0 | 0 | 2 |
| Dimpler (mean intensity) | 1 | 0 | 0 | 0 | 0 | 0 | 0 | 0 | 0 | 1 | 0 | 0 | 0 | 0 | 0 | 2 |
| Average tonic component of EDA signal | 0 | 1 | 1 | 0 | 0 | 0 | 0 | 0 | 0 | 0 | 0 | 0 | 0 | 0 | 0 | 2 |
| Cheek raiser (mean presence) | 0 | 0 | 1 | 0 | 1 | 0 | 0 | 0 | 0 | 0 | 0 | 0 | 0 | 0 | 0 | 2 |
| Variance of gaze-angle in x-direction | 0 | 0 | 1 | 0 | 0 | 0 | 0 | 0 | 0 | 1 | 0 | 0 | 0 | 0 | 0 | 2 |
| Kurtosis of skin conductance response | 0 | 0 | 1 | 0 | 1 | 0 | 0 | 0 | 0 | 0 | 0 | 0 | 0 | 0 | 0 | 2 |
| Standard deviation of interbeat interval | 0 | 0 | 1 | 0 | 1 | 0 | 0 | 0 | 0 | 0 | 0 | 0 | 0 | 0 | 0 | 2 |
| Lip tightener (mean presence) | 0 | 0 | 0 | 1 | 0 | 0 | 0 | 1 | 0 | 0 | 0 | 0 | 0 | 0 | 0 | 2 |
| Lip stretcher (mean presence) | 0 | 0 | 0 | 1 | 0 | 0 | 0 | 0 | 0 | 1 | 0 | 0 | 0 | 0 | 0 | 2 |
| Power in low frequency band of HRV | 0 | 0 | 0 | 0 | 1 | 0 | 0 | 0 | 0 | 1 | 0 | 0 | 0 | 0 | 0 | 2 |
| Mean gaze-angle in y-direction | 0 | 0 | 0 | 0 | 1 | 0 | 0 | 0 | 1 | 0 | 0 | 0 | 0 | 0 | 0 | 2 |
| Chin raiser (variance intensity) | 0 | 0 | 0 | 0 | 1 | 0 | 0 | 1 | 0 | 0 | 0 | 0 | 0 | 0 | 0 | 2 |
| Mean distance from camera | 0 | 0 | 0 | 0 | 0 | 1 | 0 | 0 | 0 | 0 | 1 | 0 | 0 | 0 | 0 | 2 |
| Mean gaze-angle in x-direction | 0 | 0 | 0 | 0 | 0 | 1 | 0 | 1 | 0 | 0 | 0 | 0 | 0 | 0 | 0 | 2 |
| Maximum of EDA signal | 0 | 0 | 0 | 0 | 0 | 0 | 1 | 0 | 1 | 0 | 0 | 0 | 0 | 0 | 0 | 2 |
| Median heart rate | 0 | 0 | 0 | 0 | 0 | 0 | 0 | 0 | 0 | 1 | 0 | 0 | 1 | 0 | 0 | 2 |
| Lip stretcher (mean intensity) | 0 | 0 | 0 | 0 | 0 | 0 | 0 | 0 | 0 | 1 | 0 | 1 | 0 | 0 | 0 | 2 |
| Lips part (mean intensity) | 0 | 0 | 0 | 0 | 0 | 0 | 0 | 0 | 0 | 1 | 1 | 0 | 0 | 0 | 0 | 2 |
| Mean pitch rotation | 0 | 1 | 0 | 0 | 0 | 0 | 0 | 0 | 0 | 0 | 0 | 0 | 0 | 0 | 0 | 1 |
| Minimum heart rate | 0 | 0 | 1 | 0 | 0 | 0 | 0 | 0 | 0 | 0 | 0 | 0 | 0 | 0 | 0 | 1 |
| Mean location of head on x-axis | 0 | 0 | 1 | 0 | 0 | 0 | 0 | 0 | 0 | 0 | 0 | 0 | 0 | 0 | 0 | 1 |
| Median amplitude of EDA signal | 0 | 0 | 1 | 0 | 0 | 0 | 0 | 0 | 0 | 0 | 0 | 0 | 0 | 0 | 0 | 1 |
| Blink (mean intensity) | 0 | 0 | 1 | 0 | 0 | 0 | 0 | 0 | 0 | 0 | 0 | 0 | 0 | 0 | 0 | 1 |
| Mean amplitude of EDA signal | 0 | 0 | 1 | 0 | 0 | 0 | 0 | 0 | 0 | 0 | 0 | 0 | 0 | 0 | 0 | 1 |
| Area under the curve of EDA signal | 0 | 0 | 1 | 0 | 0 | 0 | 0 | 0 | 0 | 0 | 0 | 0 | 0 | 0 | 0 | 1 |
| Power in low frequency band of HRV | 0 | 0 | 1 | 0 | 0 | 0 | 0 | 0 | 0 | 0 | 0 | 0 | 0 | 0 | 0 | 1 |
| Jaw drop (variance intensity) | 0 | 0 | 0 | 1 | 0 | 0 | 0 | 0 | 0 | 0 | 0 | 0 | 0 | 0 | 0 | 1 |
| Lip corner depressor (mean presence) | 0 | 0 | 0 | 0 | 1 | 0 | 0 | 0 | 0 | 0 | 0 | 0 | 0 | 0 | 0 | 1 |
| Mean interbeat interval | 0 | 0 | 0 | 0 | 1 | 0 | 0 | 0 | 0 | 0 | 0 | 0 | 0 | 0 | 0 | 1 |
| Variance of gaze-angle in y-direction | 0 | 0 | 0 | 0 | 1 | 0 | 0 | 0 | 0 | 0 | 0 | 0 | 0 | 0 | 0 | 1 |
| Inner brow raiser (variance intensity) | 0 | 0 | 0 | 0 | 1 | 0 | 0 | 0 | 0 | 0 | 0 | 0 | 0 | 0 | 0 | 1 |
| Upper lip raiser (mean presence) | 0 | 0 | 0 | 0 | 1 | 0 | 0 | 0 | 0 | 0 | 0 | 0 | 0 | 0 | 0 | 1 |
| Ratio between power in high and low frequency bands of HRV | 0 | 0 | 0 | 0 | 1 | 0 | 0 | 0 | 0 | 0 | 0 | 0 | 0 | 0 | 0 | 1 |
| Average phasic driver component of EDA signal | 0 | 0 | 0 | 0 | 1 | 0 | 0 | 0 | 0 | 0 | 0 | 0 | 0 | 0 | 0 | 1 |
| Root mean squared of successive differences in HRV | 0 | 0 | 0 | 0 | 1 | 0 | 0 | 0 | 0 | 0 | 0 | 0 | 0 | 0 | 0 | 1 |
| Upper lip raiser (mean intensity) | 0 | 0 | 0 | 0 | 1 | 0 | 0 | 0 | 0 | 0 | 0 | 0 | 0 | 0 | 0 | 1 |
| Variance in yaw head rotation | 0 | 0 | 0 | 0 | 1 | 0 | 0 | 0 | 0 | 0 | 0 | 0 | 0 | 0 | 0 | 1 |
| Lip corner puller (variance intensity) | 0 | 0 | 0 | 0 | 0 | 1 | 0 | 0 | 0 | 0 | 0 | 0 | 0 | 0 | 0 | 1 |
| Outer brow raiser (variance intensity) | 0 | 0 | 0 | 0 | 0 | 0 | 0 | 1 | 0 | 0 | 0 | 0 | 0 | 0 | 0 | 1 |
| Mean yaw head rotation | 0 | 0 | 0 | 0 | 0 | 0 | 0 | 1 | 0 | 0 | 0 | 0 | 0 | 0 | 0 | 1 |
| Chin raiser (mean presence) | 0 | 0 | 0 | 0 | 0 | 0 | 0 | 1 | 0 | 0 | 0 | 0 | 0 | 0 | 0 | 1 |
| Chin raiser (mean intensity) | 0 | 0 | 0 | 0 | 0 | 0 | 0 | 0 | 0 | 1 | 0 | 0 | 0 | 0 | 0 | 1 |
| Skewness of EDA signal | 0 | 0 | 0 | 0 | 0 | 0 | 0 | 0 | 0 | 1 | 0 | 0 | 0 | 0 | 0 | 1 |
| Average phasic driver component of EDA signal | 0 | 0 | 0 | 0 | 0 | 0 | 0 | 0 | 0 | 1 | 0 | 0 | 0 | 0 | 0 | 1 |
| Skewness of interbeat interval | 0 | 0 | 0 | 0 | 0 | 0 | 0 | 0 | 0 | 1 | 0 | 0 | 0 | 0 | 0 | 1 |
| Median interbeat interval | 0 | 0 | 0 | 0 | 0 | 0 | 0 | 0 | 0 | 1 | 0 | 0 | 0 | 0 | 0 | 1 |
| Nose wrinkler (variance intensity) | 0 | 0 | 0 | 0 | 0 | 0 | 0 | 0 | 0 | 1 | 0 | 0 | 0 | 0 | 0 | 1 |
| Area under the curve of heart rate | 0 | 0 | 0 | 0 | 0 | 0 | 0 | 0 | 0 | 0 | 0 | 1 | 0 | 0 | 0 | 1 |
| Mean location of head on y-axis | 0 | 0 | 0 | 0 | 0 | 0 | 0 | 0 | 0 | 0 | 0 | 0 | 1 | 0 | 0 | 1 |
| Mean roll head rotation | 0 | 0 | 0 | 0 | 0 | 0 | 0 | 0 | 0 | 0 | 0 | 0 | 1 | 0 | 0 | 1 |
| Brow lowerer (variance intensity) | 0 | 0 | 0 | 0 | 0 | 0 | 0 | 0 | 0 | 0 | 0 | 0 | 1 | 0 | 0 | 1 |

**Table A7:** Confusion matrices for arousal scores of the experts (predicted class) on the full set of presented recordings in relation to the scores provided by participants (true class).

| **Arousal** |  |  | *True class* |  |  |  |
| --- | --- | --- | --- | --- | --- | --- |
|  | Expert 1 | | Expert 2 | | Expert 3 | |
| *Predicted class* | *High* | *Low* | *High* | *Low* | *High* | *Low* |
| *High* | **76** | **105** | **95** | **136** | **83** | **113** |
| *Low* | **50** | **75** | **31** | **44** | **43** | **67** |

**Table A8:** Confusion matrices for valence scores of the experts (predicted class) on the full set of presented recordings in relation to the scores provided by participants (true class).

| **Valence** |  |  | *True class* |  |  |  |
| --- | --- | --- | --- | --- | --- | --- |
|  | Expert 1 | | Expert 2 | | Expert 3 | |
| *Predicted class* | *High* | *Low* | *High* | *Low* | *High* | *Low* |
| *High* | **74** | **91** | **80** | **85** | **111** | **54** |
| *Low* | **50** | **91** | **50** | **91** | **74** | **67** |
